# The contribution of executive functions to narrative writing in fourth grade children

**DOI:** 10.1007/s11145-015-9558-z

**Published:** 2015-03-28

**Authors:** Elise Drijbooms, Margriet A. Groen, Ludo Verhoeven

**Affiliations:** 1 Behavioural Science Institute, Radboud University Nijmegen, Montessorilaan 3, Room A04.23a, P.O. Box 9104, 6500 HE Nijmegen, The Netherlands; 2 Behavioural Science Institute, Radboud University Nijmegen, Montessorilaan 3, P.O. Box 9104, 6500 HE Nijmegen, The Netherlands

**Keywords:** Executive functions, Writing development, Narratives, Children

## Abstract

The present study investigated the contribution of executive functions to narrative writing in fourth grade children, and evaluated to what extent executive functions contribute differentially to different levels of narrative composition. The written skills of 102 Dutch children in fourth grade were assessed using a narrative picture-elicitation task. In addition, a large test battery assessing transcription skills, language skills and executive functions, was administered. The results showed that executive functions contributed both directly and indirectly to narrative composition. More specifically, analyses revealed that inhibition and updating, but not planning, contributed directly to the text length of the narrative, and indirectly, through handwriting, to the text length, syntactic complexity, and story content. The findings underscore the need to assess a variety of executive functions and support the idea that in developing writers executive functions also play a role in more complex written composition tasks, such as narrative writing.

## Introduction

One of the first and most widely accepted definitions of writing is that the act of composing a text is a goal-directed thinking process which is guided by the writer’s own growing network of goals (Hayes & Flower, 1980). Regarding writing as a goal-directed activity entails the assumption that several executive functions (EF)—mental processes involved in goal-directed activities (Miller & Cohen, 2001; Shallice, 1982)—underlie and support its execution.

Within developmental writing research, EF have mostly been conceptualized as higher-level self-regulation strategies that guide and monitor the cognitive processes in writing. In the Hayes and Flower (1980) model these are known as planning, translating, reviewing, and revising. As such, much research has adopted a pedagogical approach and has focused on training these self-regulative EF in children (e.g., Graham & Harris, 1993, 1998; Harris & Graham, 1996). According to the Simple View of Writing (Berninger & Amtmann, 2003) these EF play a limited role in the early stages of writing development due to children’s immature transcription skills and the limited capacity of working memory (WM). The model conceptualizes the writing process as consisting of two primary components, transcription and EF, that support a third component known as text generation in an environment of WM. In the model, transcription encompasses handwriting and spelling. EF include the high-level processes of planning, monitoring and revising. Text generation refers to the translation of ideas into linguistic representations at the word-, sentence-, and text-level. In developing writers, transcription contributes most to text generation, as it takes up all of the available cognitive resources in WM. In order to avoid a cognitive overload, young writers resort to a knowledge-telling strategy that includes linearly writing whatever the writer knows about a topic, with very limited involvement of higher-level EF such as planning and revising (Bereiter & Scardamalia, 1987; Graham, Harris, & Olinghouse, 2007). As writing development progresses and the cognitive load of the writing task associated with the demands of transcription decreases, it is thought that young writers gradually move towards a knowledge-transforming strategy: an increasing use of higher-level EF allows them to attend to the global structure of the text, resulting in greater overall coherence (Bereiter & Scardamalia, 1987).

Recently, researchers have begun to elaborate the idea that not only high-level EF, but also low-level EF contribute to the writing process of developing writers (Berninger & Chanquoy, 2012; Berninger & Winn, 2006). In a subsequent adaptation of the Simple View of Writing, Berninger and Winn (2006) incorporate a complex system, called supervisory attention, to account for the role of low-level EF in the executive control of the writing process. Supervisory attention is thought to enable the writer to maintain attentive during the writing task, to devote conscious attention to several metalinguistic and metacognitive subtasks, and to generate cognitive engagement necessary for effective writing performance. Berninger and Richards (2002, 2010) have consequently proposed that a panel of low-level EF constitute the underpinnings of this supervisory attention architecture. The latter then enhances the intercommunication between the sensory and motor skills, the language skills, and the high-level EF as they engage in writing processes. In all, it is argued that both the low- and high-level EF contribute to writing. Although low-level EF have previously been conceptualized as a unitary construct of supervisory attention, there is now general agreement that there exist three core low-level EF that are both intercorrelated and separable: inhibition, updating, and shifting (Diamond, 2013; Lehto, Juujarvi, Kooistra, & Pulkkinen, 2003; Miyake, Friedman, Emerson, Witzki, & Howerter, 2000). Following Diamond (2013), inhibition involves (1) the capacity to selectively attend to specific stimuli in WM while suppressing attention to other stimuli (selective attention), (2) the discipline to stay on task and complete the task despite distractors (sustained attention), and (3) the ability to inhibit prepotent responses (response inhibition). Updating refers to the ability to store and update relevant information in WM. Shifting, finally, including cognitive flexibility, involves the capacity to switch between tasks and mental sets. From these so-called low-level EF higher-level EF are built such as reasoning, problem solving and planning (Diamond, 2013). Table 1 presents an overview of these EF and their corresponding cognitive skills.

**Table 1:** Overview of executive functions and their corresponding cognitive skills

Executive functions and their corresponding cognitive skills		
Low-level EF	Inhibition	(1) The ability to selectively attend to specific stimuli while suppressing attention to other stimuli (selective attention) (2) The ability to stay on task and complete the task despite distractors (sustained attention) (3) The ability to inhibit prepotent responses (response inhibition)
	Updating	The ability to store and update relevant information in WM
	Shifting	The ability to switch between tasks and mental sets
High-level EF	Reasoning, problem-solving, planning	The ability to develop new ideas, to plan in advance, and to approach tasks in an efficient/strategic manner. Writing-specific EF: (1) planning involving both idea generation and goal-setting, (2) translating cognitive representations in linguistic symbols, (3) reviewing and revising text

The panel of low-level EF, assumed to underlie and support the high-level EF (Berninger & Richards, 2010), has received much less attention and little empirical research has used standard neuropsychological measures of EF to investigate their role in developing writers. Hooper, Swartz, Wakely, de Kruif, and Montgomery (2002) showed that low-level EF tapping initiating, set-shifting and sustaining can differentiate good and poor writers in fourth and fifth grade. A subsequent study by their group (Hooper et al., 2011) demonstrated the importance of low-level EF as early predictors of spelling and written expression in even younger (first and second grade) children. Altemeier, Jones, Abbott, and Berninger (2006) investigated the importance of low-level EF for developing reading-writing connections in third and fifth graders and found that different low-level EF contributed uniquely, depending on the specific reading-writing task and the grade level of the children. More specifically, inhibition was found to contribute most to a note-taking task, whereas a shifting measure was a strong predictor of a report-writing task. Altemeier, Abbott, and Berninger (2008) showed that the low-level EF of inhibition and shifting explained variance in spelling and written expression in third, fourth and fifth grade children. The contribution of low-level EF to written expression was, however, not easily interpreted due to the lack of a linear progression across development. The authors suggested therefore that different EF might differentially contribute to word-level versus text-level writing skills: inhibition and shifting may support word-level processing, whereas other more high-level EF, not measured in their study, may predict text-level processing.

Although these recent studies suggest that empirically measured low- and high-level EF might be more important to the early development of written language skills than previously asserted, some limitations are worth noting. Firstly, few attempts have been made to disentangle the contribution of different EF to writing in children. Some have consolidated several variables assessing EF into a single EF construct for data analysis (Hooper et al., 2011), whereas others did differentiate the contribution of different EF, but used a limited test battery to assess EF (Altemeier, Jones, et al., 2006; Altemeier, Abbott, et al., 2008). While previous research has left largely unspecified how the three low-level EF of inhibition, updating and shifting established by Miyake et al. (2000) contribute to overall writing performance, based on their nature it can be assumed that they jointly contribute to assert cognitive control during the composition of written text (Kellogg, Whiteford, Turner, Cahill, & Mertens, 2013). Inhibition might for instance play a substantial role in suppressing inappropriate lexical representations at the word-level and grammatical structures at the sentence-level, and selecting a relevant set of words and phrase structures (Kellogg et al., 2013). Shifting could be involved in supporting this process by switching between an inhibited set and a newly activated set. Writers who experience difficulties in selecting the appropriate lexical and grammatical representations may take longer to generate text, resulting in shorter texts and simpler sentences. Similarly, at the textual level, writers need to inhibit irrelevant ideas so as to focus and organize the main ideas (Altemeier, Jones, et al., 2006; Kellogg et al., 2013). The organization of ideas, in turn, involves thinking over larger stretches of space and time, and might be more easily supported by higher-level EF such as planning (Altemeier, Abbott, et al., 2008). Updating, finally, might be required in changing and manipulating the contents of WM as the writer’s thoughts develop and the text emerges. Composing a text requires building and storing a text representation in long-term memory. As composing progresses, the contents of WM need to be constantly updated to align with this stored representation. Previous representations of how far the writer has progressed in the task require updating as the writer keeps track of the position in the sentence at hand and the position in the written text as a whole (St Clair-Thompson & Gathercole, 2006).

A second limitation of previous studies on writing and EF is that a variety of measures of written outcome has been used in these studies, but none have provided an elaborated assessment of written composition, such as narrative expression. Particularly in middle to late elementary school, writing tasks shift towards text-composing and become more demanding and difficult. Such tasks require careful planning and thoughtful reflection, making EF presumably more critical to writing quality (Milch-Reich, Campbell, Pelham, Connelly, & Geva, 1999; Luo & Timler, 2008; Renz et al., 2003). It is thus important to relate EF to more complex writing tasks such as written composition.

Recent research has specified two levels of written composition: microstructure (i.e., at the local word and sentence level) and macrostructure (i.e., at the global text or discourse level; Puranik, Lombardino, & Altmann, 2008; Wagner et al., 2011). Microstructural analysis typically includes measures of productivity and complexity. Macrostructural analysis, on the other hand, refers to the overarching coherence and organization of a text and may include measures of structure (e.g., logical ordering and episode structure) and content (e.g., idea units). As shown by Table 2, these micro- and macrostructural levels correspond very closely to the levels of language at which text is generated (Whitaker, Berninger, Johnston, & Swanson, 1994; Wagner et al., 2011): the productivity factor corresponds to the word-level, the complexity factor to the sentence-level, and the macrostructural factor to the text-level. Assessment of writing performance on micro- and macrostructural levels has the potential to differentiate both interindividual and intraindividual differences in the ability to translate ideas into words, sentences and text (Whitaker et al., 1994; Wagner et al., 2011). Within developing writers themselves, the competence to translate ideas into words does not necessarily imply equal competence at the sentence- and text-level. These intraindividual differences suggest that the levels of language at which text generation occurs, might each require a different process, bearing a different cognitive cost. In view of the assumption that different low- and high-level EF may be differentially relevant to word-level versus text-level writing activities (Altemeier, Abbott, et al., 2008), an interesting question is whether the word-, sentence-, and text-level within a written composition are also regulated by different EF.

**Table 2:** Overview of levels of the written composition, their corresponding levels of language and the measures used to assess performance

Levels of composition	Levels of language	Measures
Microstructure	Word-level	Productivity
	Sentence-level	Complexity
Macrostructure	Text-level	Content, structure

To summarize, EF have been recognized as an important contributor to writing in the adult writer and recently also in the beginning writer. However, studies on EF in writing of children that used standard neuropsychological measures of low- and high-level EF are limited. Furthermore, although narrative composition constitutes an important writing activity in elementary school grades, little is known about the relationship between EF and narrative composition in the young writer (but see Hooper et al., 2002). Some of the studies that adopted a neuropsychological approach have suggested that different EF may contribute differentially to word-level and text-level writing outcomes in children (Altemeier, Jones, et al., 2006; Altemeier, Abbott, et al., 2008). As such we ask whether the word-, sentence-, and text-level aspects of a written composition are also regulated by different low- and high-level EF. The current study addresses these issues by assessing narrative writing in typically developing children in fourth grade and consequently evaluating whether individual differences in empirically measured EF can predict individual differences in narrative composition at the micro- and macrostructural level. In contrast to previous studies, a broad neuropsychological test battery is used to assess low- and high-level EF tapping inhibition, updating, shifting, and planning skills, in addition to measures of transcription and language skills. Given recent evidence of the role of EF in writing of early elementary school children, it is hypothesized that EF will also predict narrative writing skills of fourth grade children. At this age, the constraint of transcription skills is assumed to have decreased significantly, allowing for more EF to be allocated to text generation. Second, we tested the hypothesis that the micro- and the macrostructural levels of the narrative composition are affected differentially by different EF, given the different levels of language involved.

## Method

### Participants

Participants included 121 Dutch fourth grade children from four elementary schools in the Netherlands. Teachers assisted in the selection process in order to exclude children with known sensory and motor impairments and children diagnosed with dyslexia, Asperger syndrome, PDD-NOS, and/or Attention Deficit (Hyperactivity) Disorder. This resulted in the exclusion of 14 children with divergent diagnoses. Children with a nonverbal cognitive ability of at least two standard deviations below the mean were also excluded from the sample (Raven’s Coloured Progressive Matrices; Raven, 1956). This was the case for five children, resulting in a final sample of 102 children for data analysis. The sample comprised 46.1 % girls and children ranged in age from 8.6 to 11.1 years, with a mean age of 9.6 years (*SD* = 5.74 months). No information about the socio-economic status (SES) of the individual children was available. However, the children attended schools that were all situated in neighbourhoods, categorized as high SES according to The Netherlands Institute for Social Research. All participating children spoke Dutch, but 7 % of the children were bilingual in that they also spoke another language at home. To control for a possible influence of linguistic diversity on the results, we ensured that bilingual children did not perform worse on vocabulary, grammar and spelling than monolingual children. The data of all bilingual children were retained for the analyses, as no significant differences were found. The children were tested at the beginning of the school year.

Two individual sessions and two classroom sessions were administered. The measures were divided between two administration blocks: Block A and Block B. Block A included the measure of nonverbal cognitive ability, the measure of handwriting fluency, and the language measures. Block B comprised the executive function measures. The order in which the blocks were administered was then counterbalanced to minimize order effects. The classroom sessions were administered by the first author and included the spelling task (first classroom session), and the narrative writing task (second classroom session).

### The narrative task

For the purpose of this study, a picture-elicitation task—the Expression, Reception and Recall of Narrative Instrument (ERRNI; Bishop, 2004)—was used to assess children’s written narrative composition skills. The instrument consists of two parallel forms, the Beach Story and the Fish Story, that are each linked to a sequenced story of 15 pictures. In this study, the initial story-telling part of the Fish Story was used as the written narrative task. Children were each presented with the picture booklet for the Fish Story. The booklet was available to them throughout the session, so they were allowed to look at the pictures while writing. The children were instructed to take their time to look at all the pictures, after which they were asked to start writing a story. Neither the duration of composition nor the length of the narrative were imposed.

### Analysis of written narratives

All written narratives were transcribed using CLAN from CHILDES (MacWhinney, 2000). Stories were divided into T-units, or minimal terminable syntactic units: defined as an independent main clause with any subordinate clauses associated with it (Hunt, 1966). The transcripts were prepared by two transcribers. Twenty percent of the narratives were transcribed by both transcribers, so that inter-rater agreement could be calculated. Inter-rater agreement was computed for segmentation of T-units. A high level of agreement (97 %) was reached. The following measures of microstructure and macrostructure were derived from the transcripts.

#### Productivity

Text length in number of words was used as a microstructural measure of productivity. Text length was calculated by counting the number of words produced in each written narrative. This variable is automatically calculated by CLAN, and therefore does not require a reliability estimate.

#### Syntactic complexity

The Mean Length of T-unit in words (MLTUw) was used as a microstructural measure of syntactic complexity. Complexity was thus calculated by dividing the number of words produced by the number of T-units. This variable is automatically calculated by CLAN, and therefore does not require a reliability estimate.

#### Story content

Story content was used to assess the macrostructure of the narrative. Story content or content coherence refers to the degree of semantic informativeness in a text, and is a frequently used measure in narrative assessment (e.g., Bishop, 2004; Cragg & Nation, 2006). Story content of the written narratives was measured following standard ERRNI procedures. The ERRNI test contains a list of 24 main ideas that are represented in the story. These ideas overlap with components of story structure (Stein & Trabasso, 1982). Two points were awarded for each idea included in the narrative; one point was given when the idea was represented only partially, or when over-general or vague language was used to represent the idea. A maximum score of 48 could be achieved. Two raters scored the story content of 20 % of the transcripts in common to practice the scoring scheme. Disagreements were resolved through discussion. Afterwards, half of the transcripts were scored by the first rater and half by the second rater. Twenty percent of the transcripts was scored by both raters to determine inter-rater reliability. The inter-rater reliability was calculated as .94.

### Transcription skills

Transcription skills were assessed by measuring handwriting fluency and spelling skills.

*Handwriting fluency* was assessed in terms of speed by means of a standardized Dutch handwriting task (the “Systematische Opsporing van Schrijfproblemen”, Van Waelvelde, De Mey, & Smits-Engelsman, 2008). This task required children to copy a short text during 5 min. The raw score was calculated by counting the number of letters written in 5 min. Test–retest reliability is reported as .69 (Van Waelvelde, Hellinckx, Peersman, & Smits-Engelsman, 2012).

To assess *spelling* skills, a standardized Dutch spelling task, the “PI-dictee” (Geelhoed & Reitsma, 1999), was administered. This task required children to spell isolated words with increasing difficulty. Words were presented in sentences, and children were asked to write down the repeated word from each sentence. The raw score was the number of words spelled correctly (max. score = 135). Test–retest reliability for this task is reported as .91 (Geelhoed & Reitsma, 1999).

### Language skills

*Grammar* was assessed by measuring the Mean Length of T-unit in words (MLTUw), as an index of syntactic complexity, during an oral narrative production task. The Beach Story of the ERRNI was used to elicit the oral narrative (Bishop, 2004). This variable is automatically calculated by CLAN, and therefore does not require a reliability estimate.

Receptive *vocabulary* knowledge was measured using the Peabody Picture Vocabulary Test (PPVT-III-NL; Dunn & Dunn, 2005). Children were shown four pictures and were asked to indicate the target picture that corresponded best to the word presented orally by the experimenter. Words were presented in 12-word sets and testing was discontinued when the child missed eight or more items in a 12-item set. Raw scores were used in the analyses (max. = 204). Internal consistency reliability is reported as .95 (Dunn & Dunn, 2005).

### Executive functions

The EF tasks were chosen as specific exemplars of the three core low-level EF of inhibition, updating, and shifting (Diamond, 2013; Miyake et al., 2000). In addition, the high-level EF of planning that has previously been suggested to be associated with written language was included in the test battery as well. Where multiple tasks were used as exemplars of an EF, the underlying aim was to ensure the representation of multiple facets of that specific EF.

To assess *inhibition* four tasks were selected. The subtest Sky Search of the Test of Everyday Attention for Children (Tea-Ch Sky Search; Manly, Robertson, Anderson, & Nimmo-Smith, 1999) was administered to assess *selective attention*. This subtest required the child to circle as many pairs of identical crafts as possible on an A3 sheet with numerous pairs of crafts randomly distributed across it. To control for motor speed, a motor control version of the test was subsequently carried out. The total time in seconds needed to complete the motor control version was then subtracted from the total time needed to complete the experimental version, resulting in an attention standard score. Test–retest reliability for this task is reported as .80 (Manly et al., 1999). To measure *sustained attention*, the Letter Digit Substitution Task (LDST; Jolles, Houx, Van Boxtel, & Ponds, 1995) was used. Children were given a sheet with a key, which represented the numbers 1–9, each paired with a different letter. The test items, i.e., letters, were printed beneath the key. Children were required to replace as many letters as possible with the appropriate digit indicated by the key in 90 seconds. The number of correct substitutions made in 90 seconds was used as the raw score. Test–retest reliability for this task is reported as .88 (Jolles et al., 1995). The subtest Walk Don’t Walk (Tea-Ch Walk Don’t Walk) and the subtest Opposite Worlds (Tea-Ch Opposite Worlds) of the Tea-Ch were used to assess *response inhibition*. The Walk Don’t Walk subtest involved listening to a tape playing go tones and stop tones. During the task, the child was given an A4 sheet showing footprints on a path of 14 squares. While listening to the tape, the child was asked to mark the footprints for the go tone until the stop tone appeared. One point was awarded when children avoided the target footprint on a stop tone; a mark in the footprint constituted a failure. The number of correct items out of 20 items was the total raw score for this task. The test–retest reliability for this task is reported as .71 (Manly et al., 1999). In the subtest Opposite Worlds children were shown a sheet with a path representing the digits one and two. In the same world trial, children were asked to say the digits actually presented as quickly as possible. In the opposite world trial, children were asked to say two for the digit one and one for the digit two. The time in seconds taken to complete the opposite world trial was recorded as the score for this subtest. Test–retest reliability for this task is reported as .85 (Manly et al., 1999).

To assess *updating* skills the Wechsler Intelligence Scale for Children-IV-Integrated Digit Span subtest (WISC-IV-I Digit Span; Wechsler, 2004) was used. In the Forward Digit Span, the child was required to repeat a sequence of digits in the correct order. Each task began with a sequence that was one digit in length. The length of the sequence increased with one digit after a level had been presented twice. In the Backward Digit Span, the procedure was the same, except the child was asked to repeat the numbers in the reverse order. The raw scores were based on 1 or 0 scores for each of the two trials for each length of digit span. The total raw score for this task was calculated by combining the raw score of the Forward Digit Span (max. = 14) and the raw score of the Backward Digit Span (max. = 14), resulting in a maximum score of 28. The internal consistency reliability for this task was calculated as .78.

To assess *shifting* skills two tasks were selected. The Letter Fluency subtest from the Delis–Kaplan Executive Function System (D-KEFS-Letter Fluency; Delis, Kaplan, & Kramer, 2001) was used to tap phonemic *verbal fluency*. Letter fluency tasks entail strategic searches with word retrieval by letters requiring the ability to mentally shift between multiple subsets of words (Rende, Ramsberger, & Miyake, 2002). During this task, the child was asked to generate as many words as possible starting with a target letter within 1 min. Two trials of the phonemic verbal fluency task were administered, one with words that begin with the letter M and one with words that begin with the letter K. The raw score was calculated by adding the number of correct words in both trials. The test–retest reliability for this task is reported as .76 (Korkman, Kirk, & Kemp, 1998). The Trail Making Test from the D-KEFS (D-KEFS-TMT; Delis et al., 2001) was administered to assess *cognitive flexibility*. The task consisted of a sheet of paper over which 32 circles were distributed. The circles included both numbers (1–16) and letters (A–P) and the child was asked to draw lines to connect the circles in an ascending pattern, with the extra challenge of alternating between the numbers and letters (i.e., 1-A-2-B-3-C, etc.). The raw score was the time needed to complete this task. The test–retest reliability for this task is reported as .89 (Delis et al., 2001).

The high-level EF of *planning* was assessed by means of the Tower of London (TOL; Shallice, 1982). Children were required to build nine increasingly difficult towers with five discs corresponding to configurations represented in a stimulus book. The children were instructed to try to achieve the goal arrangement in as few moves as possible, while taking into account specific rules regarding the movement of the discs. Scores were assigned according to the number of moves needed to finish each tower. The total raw score—used in the analyses—was calculated by adding the score of each tower (max. = 30). Internal consistency reliability for this task is reported as .84 (Delis et al., 2001).

## Results

### Descriptive statistics and correlation matrix

Descriptive statistics for the measures of the written narrative, and the measures of transcription skills, language skills, and EF are reported in Table 3. The correlations between all these measures are presented in Table 4.

**Table 3:** Descriptive statistics for the measures of the written narrative, transcription skills, language skills, and executive functions

*n* = 102	Mean	SD	Min.	Max.
The written narrative				
Text length	235.37	102.53	75	560
Syntactic complexity	6.38	1.39	2.68	10.27
Story content	26.33	5.85	12	40
Transcription				
Handwriting fluency	177.00	39.53	63	260
Spelling	95.40	16.88	41	127
Language skills				
Grammar	7.67	1.34	4.86	10.66
Vocabulary	115.16	9.24	96	141
Executive functions				
Tea-Ch Sky Search	4.42	1.58	2	12.90
Tea-Ch Walk Don’t Walk	14.00	3.50	1	20
Tea-Ch Opposite Worlds	31.49	5.03	22	47
LDST	32.52	7.25	14	49
WISC-IV-I Digit Span	12.08	2.36	5	20
D-KEFS-Letter Fluency	14.57	4.29	4	28
D-KEFS-TMT	113.63	39.41	38	240
TOL	15.14	2.63	6	21

Tea-Ch = Test of Everyday Attention for Children. LDST = Letter Digit Substitution Task. WISC-IV-I Digit Span = Wechsler Intelligence Scale for Children-IV-Integrated Digit Span. D-KEFS-Letter Fluency = Delis–Kaplan Executive Function System Letter Fluency. D-KEFS-TMT = Delis–Kaplan Executive Function System Trail Making Test. TOL = Tower of London

**Table 4:** Correlations between written narrative task measures, transcription, language skills, and measures of executive functions

	1	2	3	4	5	6	7	8	9	10	11	12	13	14	15
1. Text length	1														
2. Syntactic complexity	.33**	1													
3. Story content	.53**	.44**	1												
4. Handwriting fluency	.30**	.22*	.30**	1											
5. Spelling	.27**	.24*	.17	.26**	1										
6. Grammar	.14	.44**	.23*	.05	.14	1									
7. Vocabulary	.19	.03	.26**	.11	.13	−.10	1								
8. Tea-Ch Sky Search	.27**	−.00	.14	.24*	.06	.09	.07	1							
9. Tea-Ch Walk Don’t Walk	.25*	.18	.17	.16	.22*	.12	.02	.02	1						
10. Tea-Ch Opposite Worlds	.24*	.21*	.10	.26**	.13	−.09	.02	.09	.36**	1					
11. LDST	.42**	.12	.24*	.20	.23*	.07	−.02	.13	.15	.42**	1				
12. WISC-IV-I Digit Span	.15	.22*	.14	.16	.41**	.13	−.09	.13	.18	.15	.10	1			
13. D-KEFS-Letter Fluency	.18	.07	.16	−.04	.05	.02	.06	.12	.13	.21*	.17	.15	1		
14. D-KEFS-TMT	.07	.11	.17	.19	.19	.11	.14	.14	.25*	.49**	.25*	.27**	.19	1	
15. TOL	.07	.05	.15	−.01	−.03	.07	.06	.03	.07	.03	.12	.07	−.01	.32**	1

* *p* < .05, ** *p* < .01

### Principal components analysis

In this study, the EF tasks were treated as formative measures of the EF constructs, meaning that they were seen as components of one particular EF that (jointly) define or “cause” that EF, rather than being “effects” of an underlying EF (Willoughby, Holochwost, Blanton, & Blair, 2014). As such, analyses were not conducted with the aim of revealing an underlying EF structure. To reduce and summarize the data, a principal components analysis (PCA) with orthogonal rotation (varimax) was conducted on the EF measures of the 102 subjects. Using the criteria of eigenvalues greater than one, the PCA extracted three factors. The eigenvalues and percentage of variance accounted for by the first three factors before rotation are reported in Table 5. These three factors explained 55.5 % of the total variance in the data set. The rotated factor loadings for each of the eight dependent measures are presented in Table 6. To determine factor consistency, a loading of ±.50 was used as a criterion (Tabachnick & Fidell, 2001).

**Table 5:** Eigenvalues and percent of variance for three factors

Factor	Eigenvalue	Percent of variance	Cumulative percent
1	2.32	29.0	29.0
2	1.09	13.6	42.6
3	1.03	12.9	55.5

**Table 6:** Executive function component structure identified in principal components analysis with orthogonal rotation

	Factor		
	1	2	3
Tea-Ch Walk Don’t Walk	.67	−.08	.03
Tea-Ch Opposite Worlds	.83	.12	.02
LDST	.56	.21	.06
D-KEFS-TMT	.56	.26	.52
WISC-IV-I Digit Span	.20	.49	.22
D-KEFS-Letter Fluency	.34	.50	−.25
Tea-Ch Sky Search	−.11	.83	.03
TOL	.02	.01	.91

The PCA demonstrated that the measures of EF can be consolidated into three different factors. Factor 1 includes the Tea-Ch Walk Don’t Walk and the Tea-Ch Opposite worlds, both measuring response inhibition, the LDST, measuring sustained attention, and the D-KEFS-TMT, measuring cognitive flexibility. Factor 2 includes the WISC-IV-I Digit Span, assessing updating of working memory, the Tea-Ch Sky Search, measuring selective attention, and the D-KEFS-Letter Fluency, measuring phonemic verbal fluency. Factor 3 encompasses the TOL, measuring the high-level EF of planning and strategic organization, and also the D-KEFS-TMT, measuring cognitive flexibility, loads on this factor. The PCA demonstrates some important findings. Factor 1 includes several attentional tasks, and can thus reliably be labeled Inhibition. The tasks measuring updating of working memory and selective attention both load on Factor 2, which is not surprising given their similarity in terms of neural basis (Diamond, 2013). This factor was therefore called Updating. Factor 3 distinguishes the higher-level EF of planning as a separable factor, showing a moderate intercorrelation with aspects of cognitive flexibility. This factor was therefore called Planning. The PCA showed that the tasks jointly representing the EF of shifting, D-KEFS-TMT and D-KEFS-Letter Fluency, load on all three factors. Previous studies have suggested that in children shifting might not be dissociable from inhibition and updating (van der Ven, Kroesbergen, Boom, & Leseman, 2013). In addition to the general idea that shifting highly builds on the other two core low-level EF and comes in later in development (Diamond, 2013), this might explain our finding. Overall, the PCA does not correspond entirely to the fractionation of the EF tasks as put forward by our formative measurement model, but does show a distinction between the low-level EF of inhibition and updating, and the high-level EF of planning.

The standardized factor scores (*M* = 0, *SD* = 1) derived from the PCA were subsequently computed for each child, for each factor. The factor scores were used as the variables inhibition, updating and planning in the subsequent analyses.

### Correlational analyses

Having summarized our battery of EF into three factors, the next set of analyses addressed the relationships between individual differences in writing and transcription skills, language skills and EF. Performance on writing was evaluated by assessing productivity (text length in number of words) and syntactic complexity (MLTUw) on a microstructural level, and story content (number of idea units mentioned in the text) on a macrostructural level of the written narrative. Descriptive statistics for the writing measures can be found in Table 3. Table 7 shows the correlations of the written narrative task measures with transcription, language skills and EF. These results demonstrate that on a microstructural level, text length correlates significantly with transcription skills of handwriting fluency and spelling, as well as with the factors of inhibition and updating. Microstructural syntactic complexity correlates significantly with transcription skills of handwriting fluency and spelling, as well as with grammar and with inhibition. On a macrostructural level, story content relates to handwriting fluency, to vocabulary and to grammar.

**Table 7:** Correlations of written narrative task measures with transcription, language skills and executive functions

	Microstructure		Macrostructure
	Text length	Syntactic complexity	Story content
Transcription			
Handwriting fluency	.30**	.22*	.30**
Spelling	.27**	.24*	.17
Language skills			
Vocabulary	.19	.03	.26**
Grammar	.14	.44**	.23*
EF			
Inhibition	.29**	.23*	.19
Updating	.28**	.07	.19
Planning	−.01	.05	.13

* *p* < .05, ** *p* < .01

### Regression analyses

Based on the observed correlations, three multiple regression analyses were used in order to determine the variables that predict text length, syntactic complexity and story content. Hierarchical regression analyses were used to test whether EF could improve the prediction of performance on the written narrative, after controlling for transcription and language skills. More specifically, the first step always comprised the transcription skills, whereas the second step comprised the language skills. The target set of variables, i.e., the EF, were entered in the last step so that their contribution would not be overestimated. Table 8 summarizes the outcome of these analyses.

**Table 8:** Hierarchical regression analyses predicting text length, syntactic complexity and story content of the written narrative in fourth grade children

Predictor variables	Microstructure				Macrostructure	
	Text length		Syntactic complexity		Story content	
	*R* ^2^	β	*R* ^2^	β	*R* ^2^	β
1. Transcription	.13		.09		.10	
Handwriting		.24*		.17		.28**
Spelling		.22*		.20*		.10
2. Language skills	.16		.25		.20	
Vocabulary		.14		.02		.25**
Grammar		.11		.41**		.24*
3. EF	.24		.27		.24	
Inhibition		.22*		.15		.13
Updating		.21*		−.03		.12
Planning		−.04		.00		.08

* *p* < .05, ** *p* < .01

For the prediction of text length, transcription skills accounted for 13 % of the unique variance. As can be seen from the standardized beta scores of each variable, the variance is explained by both handwriting fluency and spelling. In this analysis, language skills did not account for a statistically significant amount of variance. EF explained 8 % of unique variance after controlling for transcription and language skills. The variance is almost entirely explained by inhibition and updating. Considering syntactic complexity, transcription skills accounted for 9 % of the observed variance in syntactic complexity, with only spelling being a significant contributor. After adding the language skills, the model accounted for an additional 16 % of the observed variance. The variance is entirely explained by the variable grammar. EF did not make a significant contribution to the prediction of syntactic complexity. Finally, for story content of the written narrative, transcription skills accounted for 10 % of the variance in story content, which is entirely explained by handwriting fluency. Language skills accounted for an additional 10 % of the variance, with both vocabulary and grammar being significant predictors. Finally, none of the EF domains made a unique significant contribution to the prediction of story content.

### Path analysis

The regression analyses showed that EF did not contribute to the syntactic complexity nor to the story content of the written narratives over and above transcription skills and language skills. These findings could be interpreted in the framework of recent models of writing (e.g., Berninger & Winn, 2006), that state that in young writers, transcription skills hinder the contribution of EF to text generation, as unautomatized transcription consumes most of the available cognitive resources. However, as the contribution of EF to transcription skills is expected from the literature (Altemeier, Abbott, et al., 2008), a path analysis, using LISREL software (version 8.80, Jöreskog & Sörborn, 1996) and maximum likelihood estimation, was undertaken to test the possibility that EF do influence performance on the written narrative, with transcription skills functioning as a mediator. Language skills were not included in the model for both methodological and theoretical reasons. Methodologically, including language skills would render the ratio for the number of subjects in our sample to the number of model parameters to be estimated insufficient. Furthermore, the path analysis was conducted to test the theoretically motivated hypothesis that transcription skills mediate the relationship between EF and narrative composition. The contribution of language skills—a theoretically less likely candidate for mediation—was already explored in the regression analyses, and therefore excluded from the path analysis.

**Fig. 1:**
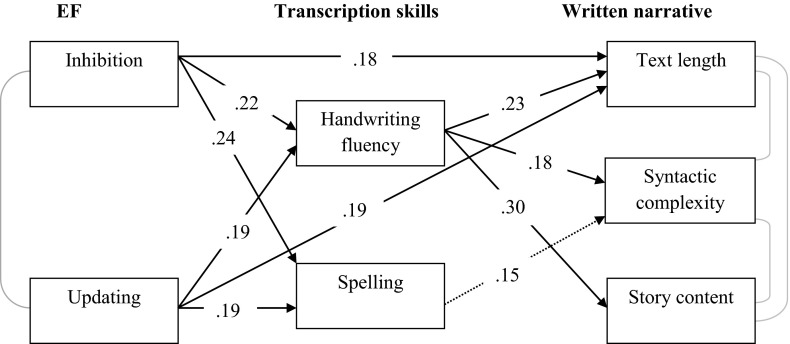
The influence of transcription skills on the relation between EF and the written narrative

The fit of the path model was evaluated by Chi square analyses and a number of goodness of fit indices. For an adequate fit, the Chi square test should exceed .05 (Ullman, 2001). For a model to be satisfactory, the goodness of fit index (GFI), the comparative fit index (CFI), the adjusted goodness of a fit (AGFI) and the normed fit index (NFI) should be greater than .90 and the root mean square error of approximation (RMSEA) lower than .08 (Jaccard & Wan, 1996; Hu & Bentler, 1999). The final model is depicted in Fig. 1. Dashed lines represent paths that are not significant. The fit of this model was satisfactory (χ^2^ = 11.01, *p* = .14, *df* = 7; RMSEA = .07, GFI = 1.00, NFI = .91, CFI = .97, AGFI = .90). The contribution of planning to the transcription and writing performance outcomes was not significant, so this EF was removed from the final model.

Figure 1 shows that inhibition and updating both directly contribute to text length, whereas they do not contribute directly to syntactic complexity or to story content. This result confirms the findings of the regression analyses previously discussed. Figure 1 also shows that inhibition and updating contribute to both handwriting fluency and spelling, and that handwriting fluency, but not spelling, contributes to text length, syntactic complexity and story content. Inhibition and updating therefore indirectly affect all writing performance outcomes. This implies that handwriting fluency mediates the relation between EF and writing performance on both the microstructural and macrostructural level.

## Discussion

The aim of the current study was to investigate the contribution of EF to narrative writing in fourth grade children. Our study adds to the existing literature in the following respects. First, unlike much research in the area of written language, a large test battery of standard neuropsychological measures of low- and high-level EF was used. Second, a developmentally relevant writing task, narrative composition, was used to assess writing skills. Finally, by focusing on the micro- and macrostructural level of the composition, this study provided the first investigation of the relationship between EF and word-, sentence-, and text-level aspects of a written composition. It was hypothesized, first, that EF would predict narrative composition, and second, that different EF would contribute differentially to the different levels of the composition.

Overall, our results show that in fourth grade children EF contribute to narrative composition in two ways. First of all, inhibition and updating contributed uniquely and directly to the text length of the written narratives, thus to word-level text generation, over and above transcription skills and language abilities. Text length in number of words is a frequently used measure of production fluency in written language and a good predictor of writing quality (e.g., Berman & Verhoeven, 2002; Wagner et al., 2011). The finding that inhibition and updating contribute directly to text length may be explained by the need to suppress inappropriate lexical representations, to select the relevant ones and to actively hold and update the representations in WM during composition. A lack of fluency in these processes may result in slower language generation, and may force the child to disrupt the writing process, leading to lower production fluency and thus to a shorter text.

Secondly, inhibition and updating contributed indirectly to text length, syntactic complexity and story content of the written narratives, i.e., to the word-, sentence-, and text-level respectively, with handwriting fluency mediating the relationship between them. It is generally acknowledged that transcription requires a large amount of cognitive resources, including EF, in developing writers (Berninger & Amtmann, 2003). More precisely, in our study the link between inhibition and updating, and handwriting fluency, might reflect the role of executive control in the coordination of multiple processes during handwriting, including motor planning, orthography, orthographic-motor integration and processing speed (Altemeier, Abbott, et al., 2008; Berninger & Amtmann, 2003). Fluent handwriting skills, in turn, free up cognitive resources that can be devoted to facilitate text generation at the word-, sentence- and text-level, affecting the quantity, complexity and content of the product (e.g., Berninger et al., 1997).

These findings also lead us to conclude that the second hypothesis—that EF would contribute differentially to the word-, sentence-, and text-level of the narrative composition—was confirmed to some degree. However, the contribution of EF to the different levels of the composition does not reside in the nature of the EF, but rather in the direct or indirect way in which they exert their influence. The word-level of the composition results to be the only level that is directly regulated by EF. Previously, Altemeier, Abbott, et al. (2008) concluded that low-level EF explain variance in word-level writing processes such as spelling. The contribution of inhibition and updating to the text length of the narrative composition in our study suggests that the word-level of a written composition taps some of the same cognitive processes as those involved in word-level literacy skills such as spelling. By contrast, the sentence- and text-level of the composition only benefit indirectly from EF through handwriting. The lack of a direct contribution of EF to the sentence-level of the composition corresponds to the idea that young writers, using a serial, knowledge-telling strategy make very few executive decisions about how ideas can be arranged into hierarchical syntactic constructions (Perfetti & McCutchen, 1987). They tend to use the first linguistic expression that occurs to them to frame their ideas, without being concerned about shaping the linguistic expression in response to discourse demands (McCutchen & Perfetti, 1982). Similarly, at the textual level, young writers’ main concern is what to say next, rather than a concern for how to fit every new idea into the overall coherence of the text (Bereiter & Scardamalia, 1987). Ideas are linearly retrieved from memory and readily translated into sentences, without shaping or adjusting these ideas to the reader’s needs. The syntactic skills and attendance to the coherence of the text have thus not come under the executive control that writing requires. The use of this knowledge-telling strategy is said to be related to the high cognitive demands of handwriting (Bereiter & Scardamalia, 1987). It could thus be hypothesized that attending to the complexity of the sentence level and the coherence of the text level will come more directly under executive control, once children grow older and automatize their handwriting. Overall, our finding of a mediating role of handwriting fluency subscribes to the idea that the impact of transcription skills on children’s writing is not limited to the early grades of elementary school but extends into the intermediate grades (Wagner et al., 2011; Graham, 1997).

Inhibition and updating showed an equal pattern of contribution to the narrative composition—direct to the word-level, and indirect to the word-, sentence-, and text-level. Given the assumption that high-level EF such as planning support text-level processing in both reading comprehension and written expression (Altemeier, Jones, et al., 2006; Altemeier, Abbott, et al., 2008), it is striking that we did not find any contribution of planning skills to any of the levels of the narrative composition. Several explanations come to mind. First, planning is a complex higher-order cognitive skill that develops particularly late in childhood and undergoes a final growth spurt during the beginning of adolescence (Anderson, Anderson, Northam, Jacobs, & Catroppa, 2001; Welsh & Pennington, 1988). It might be that the fourth grade children in our sample have not yet developed their planning skills sufficiently so as to be able to devote their capacity to the thinking processes involved in written composition. Second, developing writers generally show little or no planning and goal-directed behaviour during composition (McCutchen, 1988; Scardamalia & Berciter, 1986). The idea is that children, who have not automatized their handwriting skills, can dedicate few cognitive resources to higher-level processes of planning. Given the considerable role of handwriting fluency in our sample, this explanation seems to be valid to explain the lack of contribution of planning skills to written composition. However, an indirect contribution through handwriting skills was not found either. Moreover, Altemeier, Jones, et al. (2006) did find that planning skills, as assessed by a Tower Task, contributed to expository written composition in third graders. It may therefore be that the predictive ability of planning in middle elementary school depends on the task at hand. The narrative picture-elicitation task used in our study may trigger some sort of linear storytelling: children linearly retrieve information from the pictures, with each picture serving as a stimulus for the next idea. The presence of the pictures may reduce the need for planning skills, as the structure and content of the story are more readily available to the writer. In this respect, the picture-elicitation task might have enhanced young writers’ tendency to adopt a knowledge-telling strategy, encompassing local planning with each preceding written sentence serving as a stimulus for conducting the next search of long-term memory (Bereiter & Scardamalia, 1987). Further research is needed to see whether planning skills do contribute uniquely to open-ended narrative composition, which might encourage the use of planning skills to a larger extent. Similarly, other genres such as argumentative and expository writing call upon more complex and effortful knowledge transformation skills, and require highly demanding cognitive operations (Van Hell, Verhoeven, & Van Beijsterveldt, 2008). This might lead to a higher involvement of EF, particularly of planning as a high-level EF. In this respect, future studies might examine the role of low- and high-level EF in genres other than narrative writing.

In general, our results support the idea that EF play a role in the writing of developing writers. Moreover, our study found that EF influence narrative writing, both directly, and indirectly through handwriting skills. Furthermore, the study emphasizes the importance of assessing many different low- and high-level EF with standard neuropsychological measures. Previous writing research generally conceptualized EF as self-regulating control processes necessary for implementing planning, revising and reviewing strategies. Accordingly, EF were mostly investigated by targeting these strategies in intervention studies on self-regulation. We argue that those higher-level cognitive processes cannot account for all the EF involved in writing, and that it is necessary to assess a variety of EF, including low-level EF with standard neuropsychological measures, in order to acquire more insight into the contribution of EF to writing outcome in developing writers.

Our study has some limitations that are worth mentioning. First, only one grade in writing development was discussed. Further longitudinal research is needed to examine how the contribution of EF to narrative writing changes as children move into the late elementary grades, particularly with respect to different low-level and high-level EF involved. As transcription skills become fluent with age and have less impact on cognitive load (Berninger & Winn, 2006), EF might exert a stronger influence on the written product. Consequently, it could for instance be hypothesized that in older children and more mature writers, inhibition and updating contribute directly and independently of handwriting to the sentence- and text-level of the written composition. Fourth grade is, however, a transitional grade in literacy development (Fitzgerald & Shanahan, 2000) and thus we believe that it provides a good, initial stage of development for the study of written composition.

Second, our study offers the first attempt to relate EF to micro- and macrostructural levels of narrative composition. However, measures of micro- and macrostructure can be manifold, and here only three measures—productivity, complexity and story content—were included. Other components of micro- and macrostructure, such as lexical diversity—involving semantic knowledge—or logical ordering of ideas—requiring advanced reasoning skills—might tap different cognitive and language abilities and thus change the dynamics encountered in this study. Relatedly, further research is needed to determine the predictive ability of EF when different writing outcomes are considered.

Third, our study focused on writing a narrative by hand. The results emphasize that fluent handwriting skills are essential for writing development, as non-automatized transcription skills are cognitively demanding and may constrain the use of EF. Nowadays, however, the use of computers in classrooms has become universal and children increasingly often produce their written texts by typing. Typing involves less-complex motor processes than handwriting, and is therefore considered to be less cognitively demanding (Quinlan, 2004). Following this idea, we might expect EF to contribute more strongly and more directly to narrative writing, if the writing task is performed on a computer. More specifically, with typing requiring little cognitive effort, more cognitive resources could be allocated to EF to guide and monitor text generation. This premise remains to be studied, however, as some recent studies demonstrate that the cognitive advantage of typing only holds for fluent typists. A lack of typing automaticity substantially affects the quality of a composition, as much as non-fluent handwriting does (e.g., Christensen, 2004; Connelly, Gee, & Walsh, 2007). In much the same way as in handwriting, it thus seems to be critical that typing skills are developed early. Nevertheless, an interesting direction for future research could be to empirically investigate this premise and explore the contribution of EF to different levels of the written text, produced by typing on a word-processor.

Finally, two potential methodological limitations affect the current study. As we adhered to a formative approach to EF, our analyses were not primarily intended to reveal the underlying structure of EF. The large test battery of EF tests was reduced by means of a PCA. Although the three resulting factors correspond relatively well to components of EF previously identified (Diamond, 2013; Miyake et al., 2000), PCA is above all a dimension reduction technique. The interpretation of the underlying EF structure should thus be considered cautiously. Furthermore, small sample size is a limiting factor that constrained us to opt for a path analysis rather than a structural equation model. Sensitivity to error is a specific limitation of path analysis. To accommodate this limitation, construct validity was maximized through the use of established measures, and the reliabilities of the measures were carefully inspected. Nevertheless, the reported impact among variables should be considered as suggestive, and further research with a bigger sample size is warranted.

In conclusion, this study contributes to the limited empirical research on EF in young writers, showing that EF contribute both directly and indirectly to narrative writing, a previously neglected writing outcome in this regard. Specifically, inhibition and updating directly contributed to text length, and indirectly via handwriting fluency to text length, syntactic complexity, and story content. Our study also shows that studying different levels of a written composition has the potential to relate cognitive cost in writing to different word-, sentence-, and text-level aspects of a written composition. Although writing researchers are showing an increased interest in EF as neuropsychological predictors of writing skills, much remains to be learned about how EF support young writers in the process of becoming expert writers.
